# Retraction: Controlling air pollution by lowering methane emissions, conserving natural resources, and slowing urbanization in a panel of selected Asian economies

**DOI:** 10.1371/journal.pone.0311785

**Published:** 2024-10-14

**Authors:** The PLOS ONE Editors

This article [1] was identified as one of a series of submissions for which PLOS has concerns about adherence to journal policies on authorship and systematic manipulation of the publication process.

In addition, the corresponding author stated that the dataset downloaded from the World Development Indicator Database and used in this study is no longer available.

A member of the Editorial Board reviewed the article and data from the version of the World Development Indicator Database available at the time of post-publication follow-up [2]. They noted concerns including inadequate discussion of the choice of proxy measures, missing observations for many variables, and insufficient information about the handling of missing observations which may affect the results of the analyses. These issues call into question the validity and reliability of the reported results.

The *PLOS ONE* Editors retract this article because of the above concerns. We regret that the issues were not identified prior to the article’s publication.

KZ did not agree with the retraction and stands by the article’s findings. SH, ML, KH, SH, BU, and MA either did not respond directly or could not be reached.
